# Development of PEGylated PLGA nanoparticle for controlled and sustained drug delivery in cystic fibrosis

**DOI:** 10.1186/1477-3155-8-22

**Published:** 2010-09-24

**Authors:** Neeraj Vij, Taehong Min, Rhul Marasigan, Christopher N Belcher, Steven Mazur, Hong Ding, Ken-Tye Yong, Indrajit Roy

**Affiliations:** 1 Department of Pediatric Respiratory Sciences, Johns Hopkins University School of Medicine, Baltimore, 21287, USA; 2 Institute of NanoBioTechnology, Johns Hopkins University, Baltimore, 21218, USA; 3 Department of Chemistry, State University of New York, Buffalo, 14260, USA

## Abstract

**Background:**

The mutation in the cystic fibrosis transmembrane conductance regulator (CFTR) gene results in CF. The most common mutation, ΔF508-CFTR, is a temperature-sensitive, trafficking mutant with reduced chloride transport and exaggerated immune response. The ΔF508-CFTR is misfolded, ubiquitinated, and prematurely degraded by proteasome mediated- degradation. We recently demonstrated that selective inhibition of proteasomal pathway by the FDA approved drug PS-341 (pyrazylcarbonyl-Phe-Leuboronate, a.k.a. Velcade or bortezomib) ameliorates the inflammatory pathophysiology of CF cells. This proteasomal drug is an extremely potent, stable, reversible and selective inhibitor of chymotryptic threonine protease-activity. The apprehension in considering the proteasome as a therapeutic target is that proteasome inhibitors may affect proteostasis and consecutive processes. The affect on multiple processes can be mitigated by nanoparticle mediated PS-341 lung-delivery resulting in favorable outcome observed in this study.

**Results:**

To overcome this challenge, we developed a nano-based approach that uses drug loaded biodegradable nanoparticle (PLGA-PEG^PS-341^) to provide controlled and sustained drug delivery. The *in vitro *release kinetics of drug from nanoparticle was quantified by proteasomal activity assay from days 1-7 that showed slow drug release from day 2-7 with maximum inhibition at day 7. For *in vivo *release kinetics and biodistribution, these drug-loaded nanoparticles were fluorescently labeled, and administered to C57BL6 mice by intranasal route. Whole-body optical imaging of the treated live animals demonstrates efficient delivery of particles to murine lungs, 24 hrs post treatment, followed by biodegradation and release over time, day 1-11. The efficacy of drug release in CF mice (*Cftr^-/-^*) lungs was determined by quantifying the changes in proteasomal activity (~2 fold decrease) and ability to rescue the *Pseudomonas aeruginosa *LPS (*Pa*-LPS) induced inflammation, which demonstrates the rescue of CF lung disease in murine model.

**Conclusion:**

We have developed a novel drug delivery system to provide sustained delivery of CF "correctors" and "anti-inflammatories" to the lungs. Moreover, we demonstrate here the therapeutic efficacy of nano-based proteostasis-modulator to rescue *Pa-LPS *induced CF lung disease.

## Background

The cystic fibrosis transmembrane conductance regulator (CFTR) encodes a cAMP regulated chloride channel that is retrieved (25% wild type and 99% of ΔF508-mutated) from the endoplasmic reticulum (ER) during translation and folding, and targeted to the proteasome for premature degradation[1]. Alteration of the intracellular fate of mutant CFTR by intervening the protein processing and/or proteolytic pathway has shown promise for treating CF but selective inhibition of proteostatsis demands the controlled release of optimal amounts of drug overtime. The latest fast track FDA approval of first proteasome inhibitor drug, PS-341 for treatment of refractory multiple myeloma [2-4] has initiated the examination of protein catabolism for potential therapeutic intervention in several protein processing disorders. PS-341 (pyrazylcarbonyl-Phe-Leu-boronate) is an extremely potent, stable, reversible and selective inhibitor of chymotryptic threonine protease activity[2]. PS-341 showed encouraging results when employed in hematological cancers and solid tumors by selectively inducing apoptosis in inflammatory cancer cells while normal cells recover from proteasome inhibition [5]. Proteasome inhibitors were recently shown to have dual therapeutic importance in pharmaco-gene therapy of CF airway[6]. In this study, proteasome inhibitors- LLnL and doxorubicin enhanced the CFTR gene delivery and hence CFTR-mediated short-circuit currents. Moreover, these proteasome inhibitors were also effective in suppressing functional epithelial sodium channel (ENaC) activity and currents independent of CFTR vector administration [6]. We found that PS-341 is highly selective chymotryptic proteasome inhibitor that rescues ΔF508-CFTR and IκBα from proteasomal degradation[7-9] and hence inhibits NFκB-mediated, IL-8 activation[9]. This ability to ameliorate other primary aspects of CF disease pathophysiology in addition to the rescue of misfolded CFTR from proteasomal degradation is promising for CF therapeutics. A main concern in considering the proteasome as a therapeutic target is that proteasome inhibitors may affect the normal process(es).

Over the past couple of decades, the field of drug delivery has been revolutionized with the advent of nanoparticles, wherein these particles act as inert carriers for drugs and genes to target cells or tissues[10]. This has resulted in significant improvement in methods to induce drug accumulation in target tissues with subsequent reduction in non-specific effects, a major limitation encountered in conventional therapies for chronic conditions. However, along with the many advantages of nanoparticle-mediated drug delivery, some characteristic drawbacks demand additional studies to develop an ideal formulation for therapeutic. One such drawback is the persistence of the nanoparticle system in the body long after the therapeutic effect of the delivered drug has been realized. This has led to the development of biodegradable nanoparticles, particularly comprised of the polymer polylactide-coglycolide (PLGA), where the particle matrix degrades slowly *in vivo *and the by-products like lactic and glycolic acid are easily metabolized and excreted[11]. Therefore, PLGA nanoparticles, due to their ability to entrap both water-soluble and water-insoluble molecules, are in process of extensive evaluation for the delivery of drugs, genetic materials and proteins to cultured cells and experimental animals. These nanoparticulate systems are rapidly endocytosed by cells followed by release of their therapeutic payload by both passive diffusion and slow matrix degradation[12,13].

The nano-drug delivery system used here provides controlled and sustained PS-341 delivery for selective inhibition of proteasome mediated homeostatic process (proteostasis). This study was designed to standardize the toxicity and efficacy of nano-drug delivery system in both *in vitro *and *in vivo *(WT mice) systems, and evaluate the efficacy of PLGA-PEG mediated PS-341 lung delivery in controlling inflammatory CF lung disease. The long term goal of this study was to test the efficacy of the novel nano-system to control CF lung disease for future pre-clinical development of 2^nd ^generation targeted delivery system that can selectively deliver drugs to lung epithelium. Recent studies have identified several novel "correctors" and molecular targets for functional rescue of misfolded ΔF508-CFTR protein or chronic inflammatory state but the challenge is to provide sustained and controlled drug delivery to CF subjects. We are developing methods to encapsulate selected known CF correctors, potentiators and antimicrobials, in PLGA-PEG based nanoparticles to develop this nanosystem as a therapeutic delivery vehicle for variety of CF drugs. We anticipate that therapeutic development of this novel nano-based biodegradable therapeutic vehicle will have enormous applications in treatment of chronic pathophysiology of obstructive lung diseases like CF and COPD as these systems are designed to bypass the mucus barrier and slowly release the drug to the lung tissue or cell that warrants further preclinical evaluation and standardization.

## Results

### Characterization of PLGA-PEG^PS-341 ^nanoparticles

**Figure 1:**
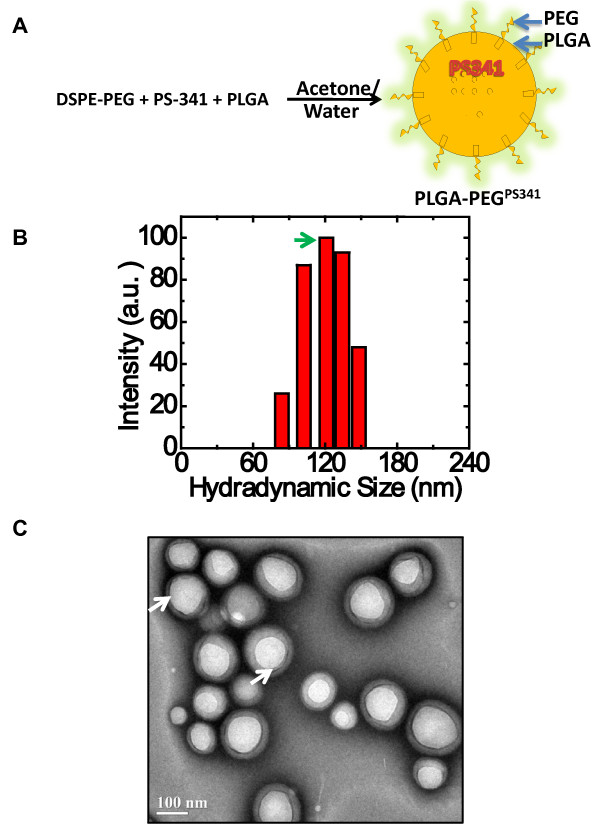
**Synthesis and characterization of PLGA-PEG^PS341 ^nanoparticles**. The PS-341 or fluorescent marker dye, nile red, loaded PLGA nanoparticles were synthesized using non-polar core of oil-in-water microemulsion technique with PEGylated phospholipid DSPE-mPEG^2000 ^as the emulsifier. Dynamic laser scattering (DLS) was employed to measure the size, distribution and colloidal stability of the PLGA-PEG^PS341 ^nanoparticles while transmission electron microscopy (TEM) was used to characterize the size and shape of the nanoparticles. **(A) **Schematic shows that PS-341 and/or nile red dye is encapsulated in PLGA nanoparticles. The hydrophobic phospholipid part of the emulsifier remains embedded in the PLGA matrix by hydrophobic interactions, whereas the hydrophilic PEG part point outwards on the nanoparticle surface, forming a polymeric brush. **(B) **The DLS results show that radius of PLGA-PEG^PS341 ^nanoparticles is 121.5 ± 15 nm (PDI = 0.106). The radius of nanoparticles varied by less than 15%, suggesting that their colloidal stability is not affected under physiological pH. **(C) **TEM shows that PLGA-PEG^PS341 ^nanoparticles are mono-dispersed, spherical and are ~200 nm in size. *DLS and TEM based size and surface characterization of nanoparticles confirms size distribution and colloidal stability of mono-dispersed particles*.

The multiple batches of PS-341 or fluorescent marker dye, nile-red, loaded PLGA nanoparticles were synthesized using non-polar core of oil-in-water microemulsion technique with PEGylated phospholipid DSPE-mPEG^2000 ^as the emulsifier. In this formulation, the hydrophobic phospholipid part of the emulsifier remain embedded in the PLGA matrix by hydrophobic interactions, whereas the hydrophilic PEG part point outwards on the nanoparticle surface, forming a polymeric brush (Fig 1A). This brush effect is implicated in the *in vivo *stability of such nanoparticles against opsonic capture by (a) shielding the high negative charge of the polymer and (b) forming a steric barrier against approaching opsonins and preventing agglomeration of nanoparticles[10]. Therefore, by using a molecule like DSPE-mPEG^2000 ^as emulsifier, we achieve both stability and PEGylation of PLGA nanoparticles. The dynamic laser scattering (DLS) results show that the average radius of PLGA-PEG^PS341 ^nanoparticles used in this study is 121.5 ± 15 nm (PDI = 0.106; Fig 1B). The diameter of nanoparticles, varied by less than 15%, suggesting that their colloidal stability is not affected under physiological pH. Transmission electron microscopy (TEM) verifies that the size of the PLGA-PEG^PS341 ^nanoparticles is ~200 nm. Moreover, data also verifies that PLGA-PEG^PS341 ^nanoparticles are mono-dispersed and spherical in shape (Fig 1C). The results were reproducible in multiple batches.

### PLGA-PEG based nano drug-delivery exhibits sustained release and activity

**Figure 2:**
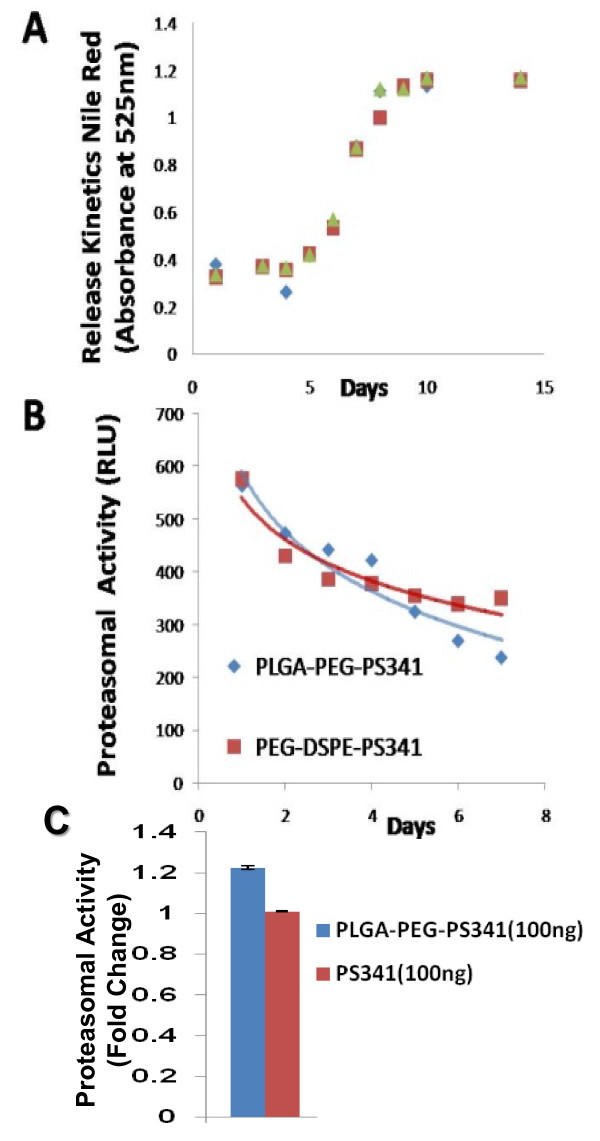
**Release kinetics of PLGA-PEG nanoparticles shows sustained release and drug activity overtime**. **A) **Release kinetics of nile red from PLGA-PEG nanoparticles (n = 3) was quantified by recording absorption of released dye at 525 nm. We observed a sinusoidal-like, sustained release of the dye from day 1 to 15, with a maximum release at day 10. Triplicate samples are shown by different symbols. **B) **We quantified the release kinetics of PS-341 from PLGA-PEG and DSPE-PEG, once daily for 7-days, using the proteasomal activity assay. We recorded proteasome inhibitory activity (Relative Luminescence Units, RLU) of room temperature incubated PLGA-PEG^PS341 ^and DSPE-PEG^PS341 ^nanoparticles for day 1 to 7, and observed more effective and sustained drug activity of PS341 from PLGA-PEG compared to DSPE-PEG. **C) **We compared the efficacy of PLGA-PEG^PS341 ^drug delivery in CFBE41o- cells as compared to PS-341 by Proteasome-Glo Chymotrypsin Cell Based Assay (Promega). We observed a significantly enhanced decrease in proteasome activity when using the PLGA-PEG mediated PS341 delivery as compared to the PS341 treatment at similar concentrations. *The PLGA-PEG nanoparticle system provides sustained release and drug activity, and enhances therapeutic effectiveness*.

**Figure 3:**
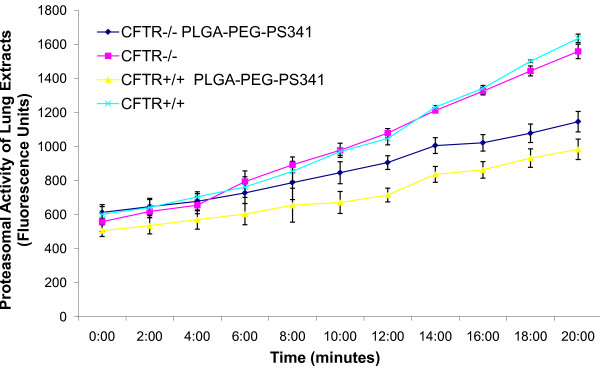
**Proteasomal activity in murine lung after proteasomal inhibition**. The proteasomes were immunoprecipitated from *Cftr^-/-^*- and *Cftr^+/+^*- mice lungs (n = 3), treated with PLGA-PEG^PS341 ^(10 μg, intranasal), and 200 μM Suc-LLVY-AMC was used as a substrate to quantify the proteasomal activity in a 96-well plate, in triplicate. Fluorescence intensities were measured at 360 nm excitation and 440 nm emission by SpectraMax Pro fluorescence plate reader. Recombinant purified proteasome was used as a positive control while no IP served as a negative control. The data shows that PLGA-PEG mediated PS341 delivery significantly inhibits the proteasomal activity (~2 fold, p < 0.01). *The data verifies the efficacy of PLGA-PEG mediated PS-341 delivery to murine lungs*.

**Figure 4:**
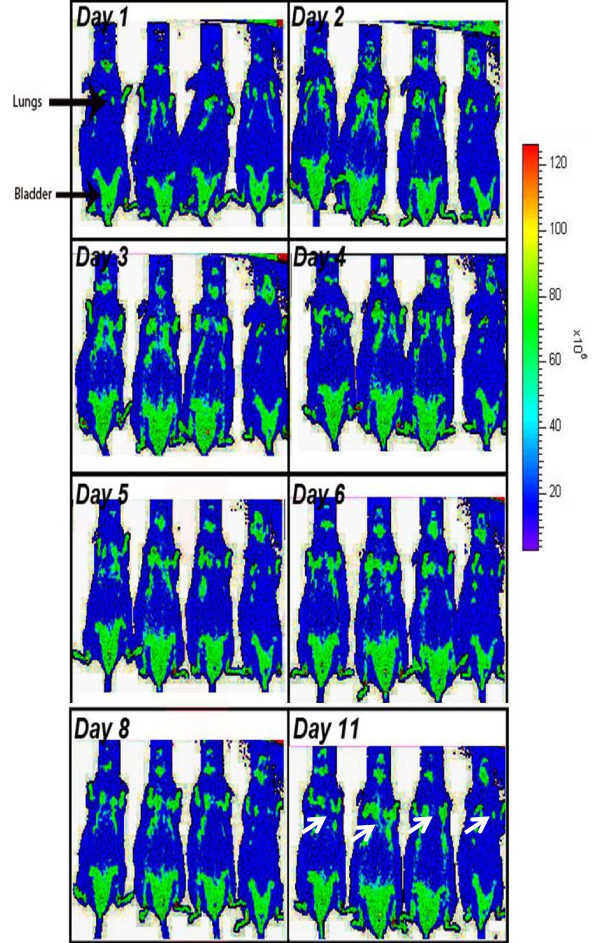
**Sustained delivery of nile red by PLGA-PEG nanoparticles**. The nile red loaded PLGA-PEG nanoparticles were insufflated in *Cftr^+/+ ^*(n = 4) mice airway. Live animals were imaged by Xenogen IVIS 200 optical imaging device (Ex 465 nm and Em 525 nm) from day 1 to 11. All animals were kept under constant supply of isoflurane using an automated anesthesia machine attached to imaging device and handled in accordance with our JHU ACUC approved animal protocol. *We observed significant amount of PLGA-PEG^PS341-NileRed ^particles in murine lungs and bladder (excreted nanoparticles) by 24 hrs and observed its sustained release from days 1 to 11 given the short half-life of the nile red*.

We determined the *in vitro *efficacy of the nanoparticle system by evaluating the release kinetics of short-lived dye (30 mins), nile red, from PLGA-PEG nanoparticles by quantifying the absorption of released dye at 525 nm. Short-lived nile red dye was selected to determine the efficacy of sustained release from nanoparticles. We observed a sinusoidal-like, sustained release of the dye from day 1 to 15, with a maximum release at day 10 (Fig 2A). Next, we quantified the release kinetics of the drug- PS-341 from PLGA-PEG *in vitro*, once every day for 7 days, using Proteasomal Activity Assay. During this experiment, we recorded proteasome inhibitory activity (Relative Luminescence Units, RLU) of room temperature incubated PLGA-PEG^PS341^- and DSPE-PEG^PS341^- (control, non-PLGA) nanoparticles for day 1 to 7 and observed sustained release of PS341 from PLGA-PEG (Fig 2B). We also observed that PLGA-PEG^PS341 ^provides more effective drug activity compared to DSPE-PEG^PS341^. Next, we compared the efficacy of PLGA-PEG^PS341 ^drug delivery in CFBE41o- cells to PS-341 treatment by Proteasome-Glo Chymotrypsin Cell Based Assay (Promega). We observed a significantly better decrease (~1.2 fold, p < 0.05) in proteasome activity when using the PLGA-PEG mediated PS341 delivery as compared to PS341 treatment (non-nanoparticle) at similar concentrations (Fig 2C). Thus, the PLGA-PEG nanoparticle enhances the drug delivery and therapeutic effectiveness. We verified these results with microscopy of PLGA-PEG^PS341/NileRed ^treated cells (described below). As a functional parameter for the *in vivo *treatment efficacy of PLGA-PEG^PS341 ^we quantified proteasomal activity in murine lung tissues. We observed significant reduction (~2 fold, p < 0.01) in proteasomal activity of *Cftr^-/-^*- and *Cftr^+/+^*- mice lungs by day-3 of intranasal PLGA-PEG^PS341 ^(10 μg) treatment (Fig 3). Next, nile red labeled PLGA-PEG nanoparticles were insufflated in *Cftr^+/+ ^*(n = 4) mice airways at indicated doses to standardize the biodistribution and release kinetics. Live animals were imaged by Xenogen IVIS 200 optical imaging device (Ex 465 nm and Em 525 nm) from day 1 to 11 under constant supply of isoflurane using an automated anesthesia machine in accordance with our JHU ACUC approved protocol. We observed significant amount of PLGA-PEG^PS341-NileRed ^particles in murine lungs by 24 hrs and observed its sustained release from days 1 to 11 given the short half-life of the nile red (Fig 4). Bladder shows the significant amounts of excreted nanoparticles demonstrating the efficient clearance of biodegradable nanoparticles overtime.

### PLGA-PEG nanoparticles mediated intracellular delivery and efficacy

**Figure 5:**
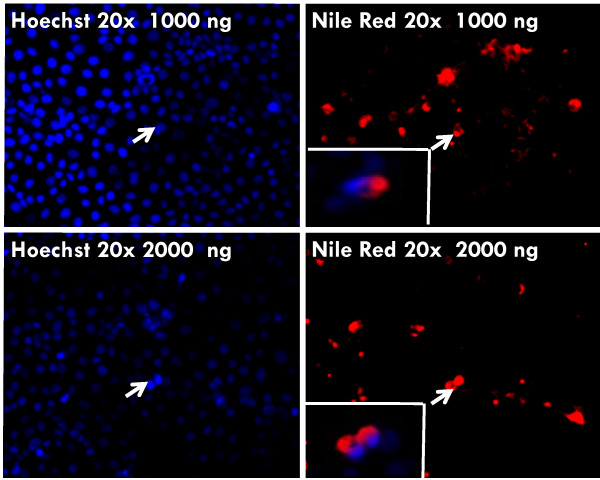
**PLGA-PEG mediated cystosolic delivery**. The indicated concentrations of PLGA-PEG^PS341-NileRed ^was added to CFBE41o- cells and incubated for 24 hours. Cells were fixed with 10% neutral buffer formalin and stained with Hoechst dye for nuclear staining. Fluorescence microscopy was used to capture images of Hoechst staining (DAPI filter) and nile red (Texas Red filter) that shows perinuclear cytosolic localization of released dye. We show the cytosolic release of nile red in perinuclear space using the PLGA-PEG nanoparticles containing 1000 or 2000 ng dye. The nile red dye added directly to the media at similar concentrations as a negative control did not show any cytosolic delivery after 24 hrs. *The data verifies the efficacy of our novel therapeutic vehicles for bronchial epithelial cell delivery*.

**Figure 6:**
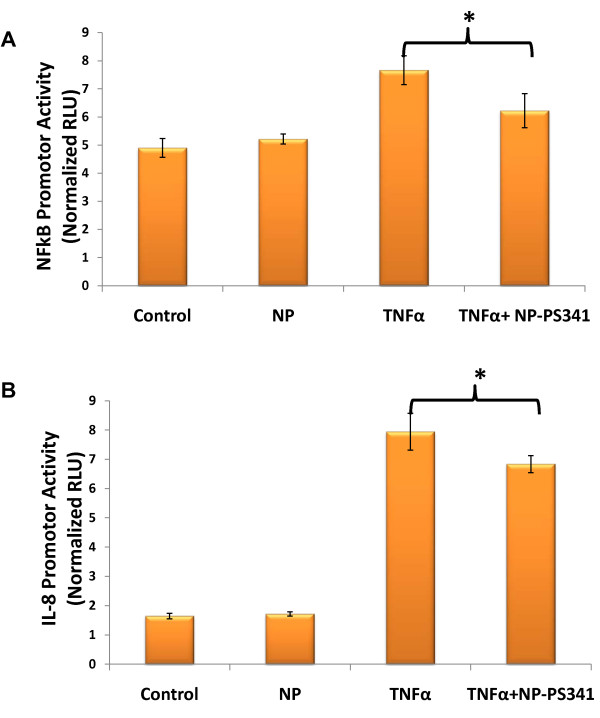
**Treatment with PLGA-PEG^PS341 ^attenuates NFκB and IL-8 promoter activities**. CFBE41o- cells were treated for 24 hours with 100 ng PLGA-PEG^PS-341 ^and transfected with NFκB, IL-8 and/or renila luciferase reporter plasmids. After six hrs of transfection, TNF-α (10 ng/ml) was used to induce proinflammatory signaling overnight. NFκB- and IL-8- firefly and renila luciferase activities were quantified using the Dual Luciferase^® ^Reporter Assay System. We observed that treatment with the 10 μl of PLGA-PEG^PS341 ^(10 ng/μl) significantly decreases TNF-α induced **A) **NFκB and **B) **IL-8 luciferase activities (*p < 0.05). Data is shown as RLU (Relative Luminescence Intensity) of firefly luciferase promoter activity normalized to renilla luciferase internal control. *The data verifies the efficacy of PLGA-PEG mediated drug delivery and activity*.

The indicated concentrations of PLGA-PEG^PS341-NileRed ^was added to CFBE41o- cells and incubated for 24 hrs followed by fluorescence microscopy to detect the nanoparticle mediated nile red delivery to CF cells. We observed the cytosolic release of nile red in perinuclear space (Fig 5) that verifies the efficacy of our therapeutic vehicle for bronchial epithelial cell delivery. For reporter assay, CFBE41o- cells were treated for 24 hours with indicated doses of PLGA-PEG^PS-341 ^after 6 hrs of NFκB or IL-8 and renila luciferase reporter plasmid transfections. The TNF-α (10 ng/ml) was used to induce proinflammatory signaling overnight. NFκB and IL-8 luciferase activity was quantified using the Dual Luciferase^® ^Reporter Assay System (Promega). We observed that treatment with the 10 μl of PLGA-PEG^PS341 ^(10 ng/μl) significantly decreased TNF-α induced NFκB (Fig 6A) and IL-8 (Fig 6B) promoter activities (*p < 0.05). The data verifies the efficacy of PLGA-PEG mediated drug delivery and NFκB inhibitory activity.

### PLGA-PEG^PS341 ^controls NFκB mediated proinflammatory response in CF lungs

**Figure 7:**
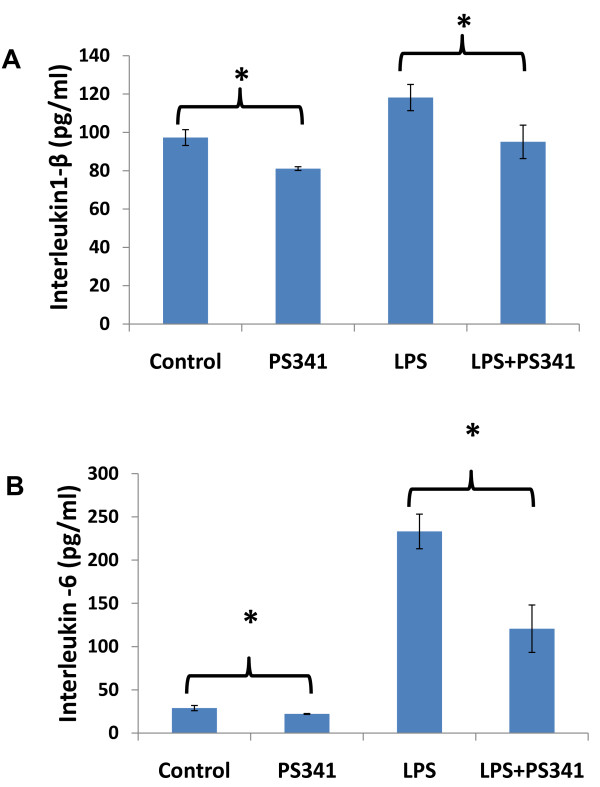
**Systemic treatment with PS-341 attenuates *Pa*-LPS induced pro-inflammatory response and neutrophil levels**. *Cftr^-/- ^*mice (n = 3, each group) were treated with *Pa*-LPS and/or PS-341 by intraperitoneal injection. Control, untreated group, was injected with 100 μl saline. The serum cytokine levels were quantified by sandwich ELISAs. We observed that treatment with the PLGA-PEG^PS341 ^decrease *Pa*-LPS induced **A) **IL1-β and **B) **IL-6 levels indicating that PS-341 can control *Pa*-LPS induced inflammatory response (*p < 0.05) if delivered efficiently to the airway. *The data indicates that PS-341 can control Pa-LPS induced inflammatory response*.

**Figure 8:**
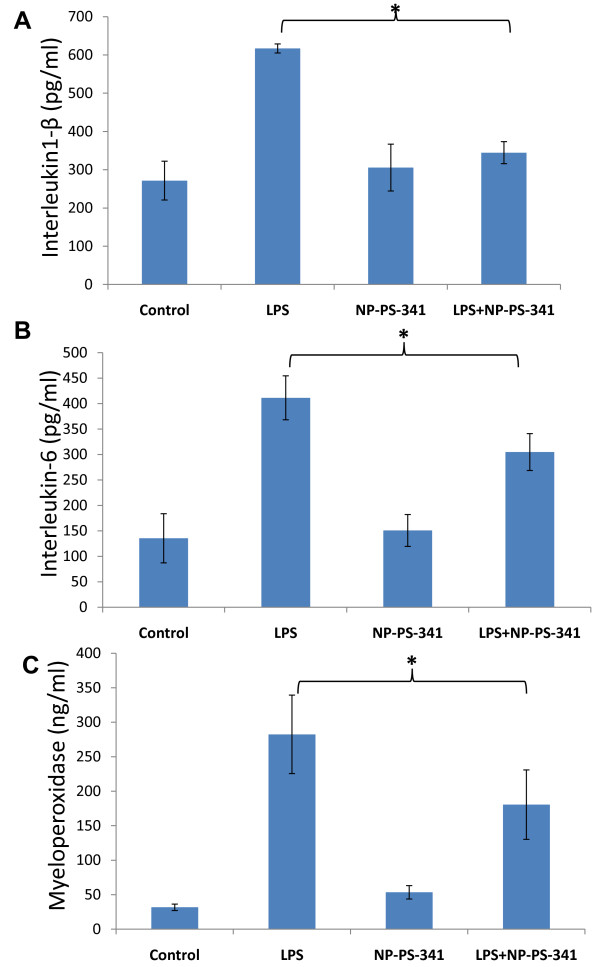
**Treatment with PLGA-PEG^PS341 ^attenuates *Pa*-LPS induced proinflammatory response and neutrophil levels**. The *Cftr^-/- ^*mice (n = 3, each group) were treated with *Pa*-LPS and/or PLGA-PEG^PS341^. Control, untreated group, was treated with 10 μl saline. The bronchoalveolar lavage fluid (BALF) cytokine and myeloperoxidase (MPO), levels were quantified by sandwich ELISAs. The treatment with the PLGA-PEG^PS341 ^significantly decreased *Pa*-LPS induced **A) **IL1-β, **B) **IL-6 and **C) **MPO levels confirming that PLGA-PEG mediated PS-341 delivery controls *Pa*-LPS induced inflammatory response and neutrophil chemotaxis (*p < 0.05). *The data verifies the efficacy of PLGA-PEG mediated PS-341 drug delivery in controlling Pa-LPS induced lung disease*.

**Figure 9:**
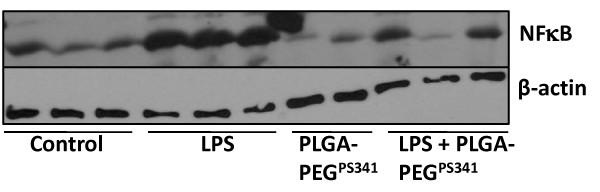
**Treatment with PLGA-PEG^PS341 ^attenuates NFκB mediated inflammatory response**. The *Cftr^-/- ^*mice (n = 3, each group) were treated with *Pa*-LPS and/or PLGA-PEG^PS341^. Control, untreated group, was treated with 10 μl saline. The lung tissue was isolated on day-3 and total protein extract was used for immunoblotting. The treatment with the PLGA-PEG^PS341 ^significantly decreases *Pa*-LPS induced NFκB levels confirming that PLGA-PEG mediated PS-341 delivery controls NFκB mediated inflammatory response in murine model. β-actin shows the equal loading. *The data verifies the efficacy of PLGA-PEG mediated PS-341 drug delivery in controlling NFκB mediated lung disease*.

To test the efficacy of PS-341 in controlling proinflammatory response, the age and sex matched *Cftr^-/- ^*mice (n = 3, each group) were injected (i.p.) with 15 mg/kg body weight *Pseudomonas aeruginosa *(*Pa*)-LPS, 24 hrs after first PS-341 treatment (0.6 mg/kg/day). Control, untreated group, was injected with 100 μl saline. Second PS-341 treatment was also given together with LPS or saline treatment and after 24 hrs, serum was collected (day-3) for ELISA. The serum cytokine levels were quantified by sandwich ELISAs. We observed that treatment with the PS-341 significantly decreased *Pa*-LPS induced IL1-β and IL-6 levels (Fig 7), demonstrating the ability of PS-341 to refrain both basal and *Pa*-LPS induced inflammatory response (*p < 0.05). Since systemic administration of PS-341 significantly inhibits the basal cytokine response, it may have immunosuppressive adverse effects. We concluded that airway delivery of PS-341 will be more effective in treating CF lung disease as compared to the intraperitoneal treatment due to increased bioavailability and reduced side effects. A main concern in considering the proteasome as a therapeutic target is that proteasome inhibitors may affect normal protein-processing machinery (proteostasis). The nano-drug delivery system used here provides a feasible alternative for controlled and sustained PS-341 delivery to lungs for selective inhibition of proteostasis to mitigate the consequences. The *Cftr^-/- ^*mice (n = 3, each group) were treated with *Pa*-LPS and/or PLGA-PEG^PS341 ^(10 μg). Control, untreated group, was treated with 10 μl saline and all mice were euthanized on day-3 as described above. The bronchoalveolar lavage fluid (BALF) cytokine and myeloperoxidase (MPO) levels were quantified by sandwich ELISAs to determine the efficacy of drug in controlling neutrophil mediated inflammatory response. We observed that treatment with the PLGA-PEG^PS341 ^significantly decreases *Pa*-LPS induced IL1-β (Fig 8A), IL-6 (Fig 8B) and MPO (Fig 8C) levels confirming that PLGA-PEG mediated PS-341 delivery controls *Pa*-LPS induced inflammatory response and neutrophil levels, *p < 0.05. The data verifies the efficacy of PLGA-PEG mediated PS-341 drug delivery in controlling *Pa*-LPS induced lung disease in CF mice. We verified that PLGA-PEG^PS341 ^treatment controls *Pa*-LPS induced NFκB protein levels (Fig 9), indicating towards its ability to control CF lung disease.

### PLGA-PEG^PS341 ^inhibits *P. aeruginosa *LPS induced CF lung disease

**Figure 10:**
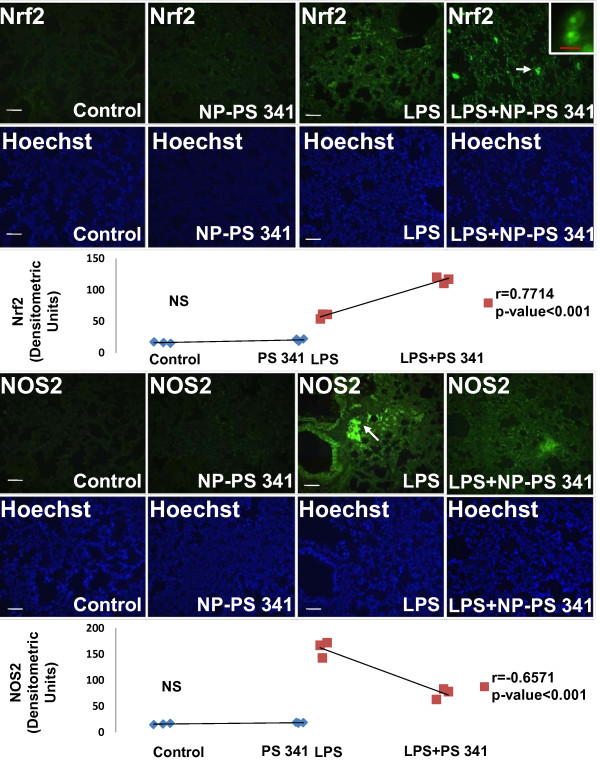
**The PLGA-PEG mediated PS-341 delivery to murine lungs controls *Pa*-LPS induced oxidative stress**. The *Cftr^-/- ^*mice (n = 3, each group) were treated with *Pa*-LPS and/or PLGA-PEG^PS341^. The PLGA-PEG^PS341 ^treated mice exhibited significant increase in Nrf2 (major antioxidant response transcription factor) expression and nuclear localization leading to decrease in LPS induced oxidative stress as seen by NOS2 immunostaining. Changes in nuclear (Nrf2) and total protein (NOS2) expression levels are shown in bottom panels (densitometry units). *The PLGA-PEG mediated PS-341 lung delivery controls Pa-LPS induced oxidative stress*. Scale: white bar = 50 μm, red bar = 10 μm.

**Figure 11:**
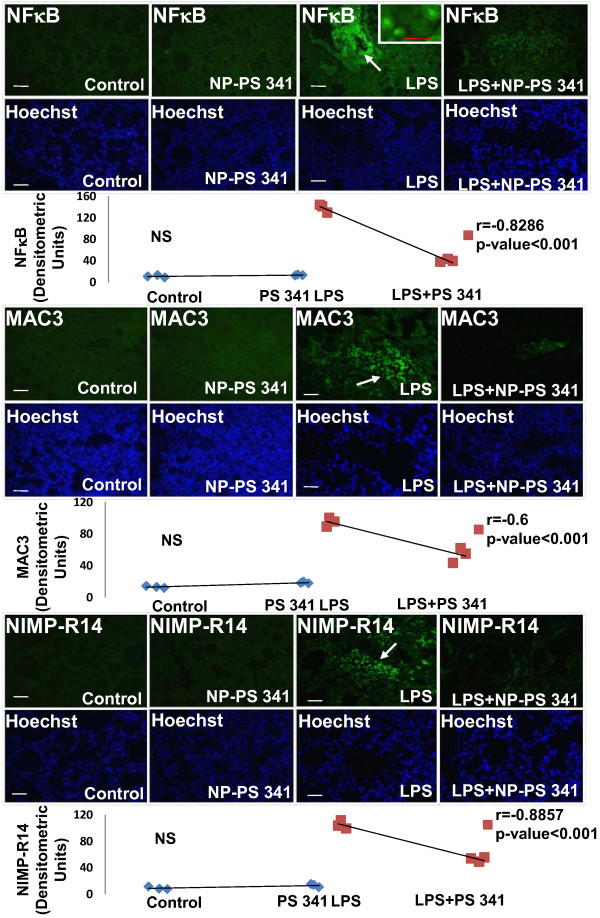
**The PLGA-PEG mediated PS-341 delivery to murine lungs controls *Pa*-LPS induced CF lung inflammation**. The *Cftr^-/- ^*mice (n = 3, each group) were treated with *Pa*-LPS and/or PLGA-PEG^PS341^. The PLGA-PEG^PS341 ^treated mice exhibited significant decrease in LPS induced NFκB expression and nuclear localization, and decline in number of inflammatory, macrophages (Mac-3^+^) and neutrophil (NIMP-R14^+^) cells. Changes in nuclear (NFκB) and total protein (Mac-3^+ ^and NIMP-R14) expression levels are shown in bottom panels (densitometry units). *The PLGA-PEG mediated PS-341 lung delivery controls Pa-LPS induced inflammation*. Scale: white bar = 50 μm, red bar = 10 μm, black = 100 μm.

**Figure 12:**
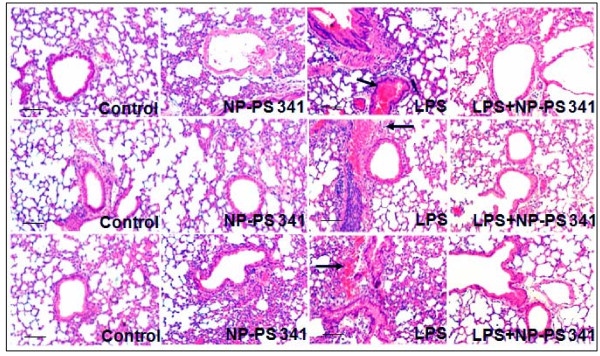
**The PLGA-PEG mediated PS-341 delivery to murine lungs controls *Pa*-LPS induced CF lung disease**. The *Cftr^-/- ^*mice (n = 3, each group) were treated with *Pa*-LPS and/or PLGA-PEG^PS341^. H&E staining verifies the rescue from *Pa*-LPS induced CF lung disease by PLGA-PEG^PS341 ^in triplicate samples. *The PLGA-PEG mediated PS-341 lung delivery controls Pa-LPS induced CF lung disease*. Scale: black = 100 μm.

The age and sex matched *Cftr^-/- ^*mice (n = 3, each group) were treated with *Pa*-LPS and/or PLGA-PEG^PS341 ^(10 μg) by insufflations and lung tissues were processed for immunostaining as described above. The PLGA-PEG^PS341 ^treated mice exhibited significant increase (day 3) in Nrf2 (major antioxidant response transcription factor) expression and nuclear localization leading to decrease in LPS induced oxidative stress as seen by NOS2 immunostaining (Fig 10). The PLGA-PEG^PS341 ^treated mice exhibited significant decrease (day 3) in LPS induced NFκB expression and nuclear localization, and decline in number of inflammatory, macrophages (Mac-3^+^) and neutrophil (NIMP-R14^+^), cells (Fig 11). H&E staining verified the rescue from *Pa*-LPS induced inflammation by PLGA-PEG^PS341 ^(Fig 12). The PLGA-PEG mediated PS341 lung delivery controls *Pa*-LPS induced inflammation and oxidative stress and has a potential to provide sustained drug delivery to control chronic CF lung disease.

## Discussion

Nanotechnology is having an increasing impact in the healthcare industry, offering unprecedented capability of not only carrying multiple diagnostic or therapeutic payloads in the same "package," but also facilitating the targeted delivery into specific sites and across complex biological barriers. The development of novel nano-systems for pulmonary gene or drug delivery may provide a convenient, noninvasive method for the administration of gene or drugs to the lungs. Such a system can also facilitate sustained site directed delivery to specific disease cell type or tissue bypassing the obstructive pathophysiological barriers. Mucous hypersecretion is a hallmark of chronic obstructive pulmonary disease (COPD) and cystic fibrosis (CF)[14]. We have previously shown that proteasomal inhibition by extremely potent, stable, reversible, and selective inhibitor of chymotryptic threonine protease activity, PS341 (Velcade/Bortezomib) rescues the CF pathophysiology of bronchial epithelial cells [9,15].

We and others have recently reported that selective inhibition of proteasome activity helps in rescue of misfolded or partially folded protein by induction of folding machinery[8,9,16-19] and it is not possible to traffic or rescue the misfolded protein by inhibiting its ubiquitination due to presence of redundant ubiquitination pathways and lack of enhanced chaperone activity. The molecular mechanisms by which proteasome inhibitors or proteostatic regulators can help in rescue of transmembrane proteins have been recently described[9,16-19]. Moreover, our recent data suggests that selective proteasome inhibition also helps in controlling chronic inflammation that will be required for treating the patients with chronic lung disease, as rescuing misfolded CFTR may not be sufficient for favorable therapeutic outcome. We confirmed that proteasome inhibition restrain the IκBα degradation[7,8] and hence NFκB-mediated, IL-8 activation[9]. PS-341 can enter mammalian cells and inhibit NFκB activation and NFκB-dependent gene expression. PS-341 is known to inhibit TNF-α-induced gene expression of the cell-surface adhesion molecules E-selectin, ICAM-1, and VCAM-1 on primary human umbilical vein endothelial cells [20,21]. In a rat model of streptococcal cell wall-induced polyarthritis [22], PS-341 attenuates the neutrophil-predominant acute phase and markedly inhibits the progression of the T cell-dependent chronic phase of the inflammatory response[20]. Clearly, this warrants further evaluation and selective delivery of this class of compounds for treatment of CF lung disease.

We evaluated the efficacy of PLGA based nano-systems for selective drug delivery. A major drawback of PLGA nanoparticles is that when formulated with the commonly used emulsifier polyvinyl alcohol (PVA), they are hydrophobic and have a high negative charge on their surface. As a result, such a system, when administered in experimental animals, is rapidly opsonized by the defense system of the body (Reticuloendothelial System, RES or Mononuclear Phagocyte System, MPS; systemic circulation or airway) [10,11]. The most common way to overcome this challenge is coating of the drug delivery system with the outer layer of polyethyleneglycol (PEG) that endow these nanoparticles with 'stealth', or RES/MPS evading properties[10]. PEGylation also increases the circulation time of the nanoparticles, thereby enhancing their propensity of accumulation in target organs or cells by passive diffusion, taking aid of the enhanced permeability and retention (EPR) effect[23]. PEG chains, covalently attached with PLGA nanoparticles using ring-opening polymerization method, results in increased residence in blood (intravenous) or airway (intranasal) and enhanced accumulation in target tissues or cells[24]. Nanoparticle mediated drug delivery presents with the added advantage of targeting the drug to specific organs or cells in the body, for example by conjugating it with a monoclonal antibody that will target the system specifically to the CF bronchial epithelial cells which over express the complementary antigen (our ongoing studies). However, until date, the use of drug loaded PLGA nanoparticles synthesized using the popular emulsifier PVA has resulted in poor *in vivo *drug delivery efficiency. It has also been found that such a formulation can never be completely purified of the emulsifier PVA, which is suspected of non-specific toxicity[25].

In order to develop an improved, clinically viable formulation of PLGA nanoparticles over existing PVA based ones, we adopted a strategy used in the synthesis of PEGylated liposomes and PEGylated immunoliposomes, and employed commercially available PEGylated phospholipids (like Distereolylphos- phatidylethanolamine-mPEG2000, or DSPE-mPEG2000) as emulsifiers[26]. Such molecules have surfactant-like properties, and spontaneously self-aggregate in aqueous solutions forming micelles[27]. We anticipate based on our studies that they can function as excellent emulsifiers for a hydrophobic polymeric system like PLGA. The DSPE-mPEG^2000 ^emulsifier provides stabilization of PLGA nanoparticles. We have designed here a novel PLGA-PEG based biodegradable therapeutic vehicle to provide sustained release of drug to the airway. The major challenge in delivery and therapeutic efficacy of nano-delivery systems in chronic obstructive airway conditions is severe inflammation and mucous hypersecretion[14,28]. Mucous hypersecretion is a hallmark of several chronic obstructive airway diseases, including COPD and CF. Distinct etiologies and inflammatory responses drive mucous hypersecretion in these diseases. In CF and COPD, the inflammatory response is neutrophilic and may be induced by infection or components in cigarette smoke. Controlling inflammation is at the root of treatment using corticosteroids, antibiotics or other available drugs in these chronic obstructive inflammatory conditions. Yet despite therapy, challenge is the sustained delivery of drugs to target cells or tissues. In spite of wide application of nano-based drug delivery systems in chronic obstructive airway diseases and variety of other pulmonary conditions like allergy, asthma, lung cancer etc, very few are tested till date[14,29-31]. To test the efficacy of our novel therapeutic drug delivery vehicle we have tested the sustained release and delivery of FDA approved proteasome inhibitor drug, PS341 in murine lungs by its ability to control *Pseudomonas aeruginosa *LPS induced CF lung disease in murine model. In this study, we determined that our PLGA-PEG drug delivery system can not only provide sustained drug release (day-3) to murine lungs but also control NFκB mediated neutrophil levels and inflammation. Our control studies using same amount of drug by insufflation, did not control neutrophil levels indicative of poor bioavailability. Our data suggest that nanoparticle mediated intranasal drug delivery helps in improving the efficacy of drug by assisting in its lung delivery and biodistribution.

The PLGA-PEG^PS341 ^provides controlled and targeted drug delivery with selective inhibition of proteasome mediated homeostatic processes (proteostasis) in lung epithelia. We observed that inhibition of the proteasome with PS341 not only rescue ΔF508-CFTR but also IκB from proteasomal degradation[7-9]; hence inhibiting the NFκB mediated- IL-8 secretion in CF[9]. We have standardized the PLGA-PEG based PS341 delivery to CF (*Cftr^-/-^*, FABP-CFTR gut corrected) murine lungs based on its ability to control *Pa*-LPS induced lung disease (Fig 8, 9, 10, 11 and 12) and inhibition of proteasomal activity (Fig 3). We found that PLGA-PEG mediated intranasal PS341 delivery, at indicated dose, results in ~2-fold inhibition of proteasomal activity in murine lungs. In addition, we have verified that intranasal delivery of fluorescently labeled PLGA-PEG^NileRed ^particles to murine lungs provide sustained release from day 1-11 (Fig 4). We observed that significant amount of particle is delivered to murine lungs by 24 hrs of inoculation. We also evaluated the release chemistry and kinetics of PLGA-PEG^PS341 ^(Fig 2A, 2B and 2C) followed by verification of functional efficacy (Figs 6, 8, 9, 10, 11 and 12).

## Conclusions

We demonstrate here the nanoparticle mediated lung delivery for treatment of CF. We anticipate that this study will have a high impact on the development of novel targeted drug-delivery therapeutics for CF and other airway diseases like COPD and asthma. The nano-drug delivery system here provides controlled and sustained PS-341 delivery for selective inhibition of proteostasis. Recent studies have identified several novel "correctors" and molecular targets for functional rescue of misfolded CFTR protein or chronic inflammatory state in CF but delivery of these drugs to CF epithelia is a challenge. Thus, further pre-clinical development of this novel nano-based biodegradable therapeutic vehicle and verification of its human (CF & COPD) mucus-penetration ability will have enormous applications in treatment of chronic pathophysiology of obstructive lung diseases.

## Materials and methods

### Cell Culture and Reagents

The CFBE41o- (cystic fibrosis bronchial epithelial cell lines, from Dr. Dieter Gruenert[32,33]) cells were maintained in MEM Earl's salt L-Glutamine (200 mM L-Glutamine) medium containing 100 units/ml penicillin, 100 μg/ml streptomycin, 0.25 μg/ml amphotericin B and 10% fetal bovine serum. MEM and other components were purchased from Invitrogen, Carlsbad, CA. TNF-α (R&D Systems Inc., Minneapolis, MN), nile red (Invitrogen), PS-341 (Millenium Pharmaceuticals, Cambridge, MA), PLGA (Avanti Polar Lipids, Alabaster, AL), DSPE-PEG^2000 ^(Avanti) and *Pseudomonas aeruginosa *LPS (Sigma, St. Louis, MO) were added to cells or injected in mice as indicated. All other common laboratory chemicals were from Sigma or Fisher Scientific.

### PLGA-PEG synthesis

We dissolved calculated amounts of PLGA and PS-341 and/or nile red in acetone and injected it in DSPE-mPEG^2000 ^emulsifier dissolved in water or PBS followed by immediate rigorous emulsification by a high power sonicator. This result in the synthesis of PEGylated nanoparticles (PNPs) of PLGA dispersed in the aqueous solution, with the water-insoluble drug (PS-341) or dye (nile red) entrapped in the hydrophobic PLGA matrix. We removed acetone by rotary vacuum evaporation and purified drug-loaded nanoparticles by ultracentrifugation followed by rigorous washing (3x) with water or PBS and resuspension in PBS.

### Transmission Electron Microscopy (TEM)

Transmission electron microscopy (TEM) was used to determine the size, shape and dispersion of PLGA-PEG^PS341 ^nanoparticles using a JEOL JEM-100cx microscope at an accelerating voltage of 100 kV. The specimens were prepared by drop-coating the sample dispersion onto a carbon-coated 300 mesh copper grid, which was placed on filter paper to absorb excess solvent.

### Dynamic laser scattering (DLS)

Dynamic laser scattering (DLS) was employed to measure the size distribution and colloidal stability of the PLGA-PEG^PS341 ^nanoparticles dispersion in water using a Brookhaven Instrument 90Plus Particle Size Analyzer at a wavelength of 633 nm and scattering angle of 90°. DLS was also used to examine the colloidal stability of nanoparticles dispersed in PBS (pH 7.4) over three days.

### Release Kinetics and Proteasome Activity Assay

Release kinetics of nile red from PLGA-PEG nanoparticles was quantified by recording absorption of released dye in resuspension buffer (PBS, 100 μl) at 525 nm using the VERSAMAX plate reader and SoftMax Pro software from molecular devices. Nanoparticle samples were aliquoted and incubated at room temperature in triplicate for indicated time points and analyzed for nile red release. We quantified the release kinetics of PS-341 from PLGA-PEG in resuspension buffer (PBS, 100 μl), once daily for a period of 7 days, using Proteasomal Activity Assay from Drug Discovery (BioMol). We recorded proteasome inhibitory activity of room temperature incubated PLGA-PEG^PS341 ^nanoparticles from day 1 to 7 following the manufacturer's protocol. We similarly quantified the efficacy of drug delivery to CFBE41o- cells by quantifying proteasomal activities of cell lysates after 24 hrs of PLGA-PEG^PS341^, PLGA-PEG (control) or PS341 treatment as indicated. We also quantified proteasomal activities in murine lungs by immunoprecipitating (IP) proteasome from lung extracts (1000 μg) using the proteasome isolation kit (Calbiochem) following the manufacturer's instructions. The 200 μM Suc-LLVY-AMC (Calbiochem) was used as a substrate to estimate chymotrypsin-like proteasomal activity in a 96-well plate. Fluorescence intensities were measured at 360 nm excitation and 440 nm emission by VERSAMax fluorescence plate reader (Molecular Devices) using the SoftMax Pro software. Recombinant purified proteasome (BIOMOL) was used as a positive control while no IP served as a negative control.

### Animal Experiments

All animal experiments were carried out in accordance with the Johns Hopkins University (JHU) Animal Care and Use Committee (ACUC) approved protocol. To induce inflammatory lung disease *in vivo*, the age (~16 weeks) and sex matched, B6- 129S6- *Cftr^-/-^*(*Cftrtm^1Kthc^*-TgN*^(FABPCFTR)^*) [34,35] inbred mice (n = 3) were treated, intratracheally (i.t., 10 μg in 100 μl PBS) or intraperitoneally (i.p., 15 mg/kg/bw in 100 μl PBS) with *Pseudomonas aeruginosa *(*Pa*)-LPS, 24 hrs post- PLGA-PEG^PS341 ^nanoparticle (intranasal, 5 μl/nostril of 1 μg/μl) or PS341 (i.p., 0.6 mg/kg/bw in 100 μl PBS for 2 days) administration. Based on a previous report [36,37] and pilot experiments on the release kinetics and *in vivo *efficacy of the drug, day-3 time point was selected for evaluating the functional efficacy of the drug. Moreover, we have previously standardized that LPS induced lung inflammation, at the selected dose, is at its peak in *Cftr^-/- ^*mice at 24 hrs[38]. Serum and total lung protein extracts were isolated at day- 3 after euthanasia in the presence of anesthesia following our JHU ACUC approved protocol. The quantification of protein levels by Western blotting of total lung protein extracts (as described below), and cytokine levels by ELISA of brochoalveolar lavage fluid (BALF)/serum (as described below) was used to identify the changes in pro-inflammatory signaling. For live animal imaging experiments, *Cftr^+/+ ^*mice insufflated with PLGA-PEG^NileRed ^nanoparticles were imaged from day 1-11 using Xenogen IVIS 200 optical imaging device (Ex 465 nm and Em 525 nm) that was directly connected to automatic anesthesia machine providing constant supply of isoflurane.

### Immunoblotting

Lung tissues were lysed by sonication (three 5 sec pulses) on ice in cold room using the T-PER (Pierce Biotech. Inc., Rockford, IL) protein lysis buffer containing protease-inhibitor cocktail (Pierce). The protein extracts were suspended in Laemmli's sample buffer (Invitrogen) containing β-mercaptoethanol (Invitrogen), resolved by 4-10% SDS-PAGE 12-well gel (lane- 1, marker; 2-4, control; 5-7, LPS; 8-9, PLGA-PEG^PS341 ^loaded in duplicate to accommodate all samples in single 12-well gel; 10-12, LPS + PLGA-PEG^PS341^) and transferred to a 0.45 μm pore size nitrocellulose membrane (Invitrogen). The β-actin (Sigma) and NFκB (Santa Cruz Biotech Inc., Santa Cruz, CA) primary antibodies, and anti-rabbit-HRP secondary antibody (Amersham, Piscataway, NJ) were used for immunoblotting.

### Immunostaining

Six-week-old mice (n = 3 per genotype) were euthanatized as described above and lungs were collected. Lung was fixed in 1 ml 10% neutral buffered formalin overnight (Fisher Scientific, Pittsburgh, PA), embedded in paraffin, sectioned, and prepared for immunostaining. Macrophages and neutrophils were immunostained with the rabbit polyclonal Mac-3 or NIMP-R14 (2 μg/ml) primary antibody (Abcam, Inc., Cambridge, UK), respectively, followed by a secondary goat anti-rat Alexa Fluor 488, 5 μg/ml (Molecular Probes, Eugene, OR) antibody. Nrf2, NOS2 and NFκB levels were similarly quantified using polyclonal antibodies from Santa Cruz Biotech Inc. Negative controls consisted of identical treatments with the omission of the primary antibody. Hoechst dye, 1 μg/ml (Molecular Probes, Invitrogen) was used for nuclear staining. The slides were then mounted (Vectashield; Vector Laboratories Inc., Burlingame, CA), and images were captured as described below. Nuclei were detected by Hoechst (Invitrogen) while H&E was used to evaluate lung morphology and inflammatory state. Images were captured by Axiovert 200 Carl Zeiss Fluorescence microscope using the Zeiss Axiocam HRC camera and Axiovision software with appropriate filter settings for FITC and DAPI. All fluorescent images were captured at room temperature with oil (63X, fluorescence) and air (20X and 40X) as the imaging medium. The magnifications for the fluorescence microscope were LD Plan- Achroplan (20X/0.40 Korr Phz), Neo Fluar (40X/0.6X Phz Korr) and Achromat (63X/1.4 oil), respectively with 1.6X optivar.

### IL-1β, IL-6 and MPO Immunoassay

At the indicated time points, BALFs or serum were collected from each mouse as reported earlier [38-40] and stored at -80C until use. BALF or serum IL-1β levels were measured using solid-phase ELISA (R&D Biosystems, Minneapolis, MN). Standards, and high and low cytokine controls were included. The plates were read at 450 nm on 96-well microplate reader (Molecular Devices, Sunnyvale, CA) using SOFT-MAX-Pro software (Molecular Devices). The mean blank reading was subtracted from each sample and control reading. The amount of substrate turnover was determined calorimetrically by measuring the absorbance, which is proportional to IL-1β concentration. A standard curve was plotted and an IL-1β concentration in each sample was determined by interpolation from standard curve. The data represents the mean ± SD of triplicate samples. The IL-6 cytokine and myeloperoxidase (MPO) levels were similarly quantified using an ELISA system (R&D Biosystems and Hycult Biotech, Canton, MA) as described before[15].

### NFκB or IL-8 Reporter Assay

CFBE41o- cells were transfected with NFκB- or IL-8- firefly luciferase promoter (pGL-2) and renila luciferase (pRLTK) control. Cells were induced with 10 ng/ml of TNF-α and/or 100 ng/ml PLGA-PEG^PS341 ^nanoparticles and luciferase activities were measured after overnight treatment. Dual-Luciferase^® ^Reporter (DLRTM) Assay System (Promega) was used to measure NFκB- or IL-8- reporter (firefly luciferase) and renila luciferase activities from CFBE41o- cell extracts. Data was normalized with internal renila luciferase control for each sample and the changes in reporter activities were calculated.

### Statistical Analysis

Representative data is shown as the mean ± SD of at least three experiments. The one-way ANOVA with a Dunnett planned comparison was run for each sample versus control. A * *p *< 0.05 was considered to have statistical significance. The murine and human microscopy data was analyzed by densitometry (Matlab R2009b, Mathworks Co.) and spearman's correlation coefficient was used to calculate the significance among the indicated groups.

## Competing interests

The authors declare that they have no competing interests.

## Authors' contributions

Conceived and designed the experiments: NV. Performed the experiments: NV, TM, RM, SM, HD, KTY and IR. Analyzed the data: NV & TM. Contributed reagents, materials and analysis tools: NV & IR. Wrote the paper: NV. Helped with the editing of the paper: KTY & IR. All authors read and approved the final manuscript.
